# The Higher the Life Satisfaction, the Better the Psychological Capital? Life Satisfaction and Psychological Capital: A Moderated Mediation Model

**DOI:** 10.3389/fpsyg.2021.772129

**Published:** 2022-04-13

**Authors:** Huanhuan Shan, Zahari Ishak, Liheng Fan

**Affiliations:** ^1^Department of Educational Psychology and Counseling, Faculty of Education, University of Malaysia, Kuala Lumpur, Malaysia; ^2^Department of Psychology, Faculty of Educational Science, Henan University, Kaifeng, China

**Keywords:** life satisfaction, psychological capital, rejection sensitive, avoidant attachment, moderated mediation model

## Abstract

This study investigates the mediator role of attachment avoidance and the moderator role of rejection sensitivity on the links between life satisfaction and psychological capital (PsyCap). This study uses the Experiences in Close Relationship Scale, Rejection Sensitive Scale, Positive Psychological Capital Scale, and Life Satisfaction Scale among 999 Chinese young adults as subjects. The results presented that life satisfaction had a significant positive predictive effect on PsyCap. Mediation analysis of this study shows that attachment avoidance mediated the association between life satisfaction and PsyCap. Furthermore, moderated mediation analysis indicated that rejection sensitivity moderates the link between life satisfaction and attachment avoidance (first-stage moderation). Compared with individuals with low rejection sensitivity, individuals with high rejection sensitivity show more attachment avoidance under low life satisfaction. This study helps understand the relationship between life satisfaction and PsyCap from the perspective of rejection sensitivity theory and attachment theory and has implications for guiding college students toward strengthening PsyCap and weakening rejection sensitivity.

## Introduction

The coronavirus disease 2019 (COVID-19) spread has led to new thinking about life and its meaning for everyone. In recent decades, there have been a growing number of studies in life satisfaction, and numerous studies have evaluated various correlations and predictors of life satisfaction. In general, life satisfaction is a cognitive evaluation of the overall quality of life based on an individual’s criteria (Shin and Johnson, 1978). As an indicator of a person’s quality of life and an important parameter to measure the quality of life of social people, life satisfaction is closely related to people’s sense of existential value. More specifically, the degree to which people are satisfied with their lives is affected by many factors, such as lockdowns at home, national identity, psychological distance, self-esteem, and leisure nostalgia, among other factors (Diener and Diener, 2009; Cho, 2020; Hamermesh, 2020; Jaspal et al., 2020; Zheng et al., 2020). Meanwhile, life satisfaction is related to a wide range of academic achievements and engagements (Suldo et al., 2006; Heffner and Antaramian, 2016). Also, an individual’s life satisfaction plays a vital role and contributes highly to mental health problems (Lombardo et al., 2018).

Life satisfaction is a vital construct in positive psychology and a critical index of subjective well-being (Gilman and Huebner, 2003). A previous study has documented that psychological capital (PsyCap) is positively linked to higher levels of life satisfaction (Bockorny and Youssef-Morgan, 2019; Datu and Valdez, 2019). PsyCap is a kind of “individual positive psychological development state,” which is malleable, developed, and promoted (Luthans et al., 2007a). PsyCap consists of four constituent elements, namely, self-efficacy (belief in self-confidence), optimism (an individual’s permanent and pervasive attribution in explanatory style for good vs. adverse events), hope (a sense of effectiveness and also persistence in aim), and resiliency (the individual’s ability to return to a positive or normal state after facing a significant setback or stressful situation) (Luthans et al., 2007b). From this perspective, individuals with a high level of life satisfaction would more likely to report more significant PsyCap and more excellent positive functioning. Thus, the following is the hypothesis.

**Hypothesis 1:** Life satisfaction is positively related to PsyCap.

However, further study is needed to reveal the mediating and moderating mechanisms underlying the links to life satisfaction on PsyCap. Attachment theory offers a dynamic approach, which provides that individuals with different attachment styles have different levels of life satisfaction (Bowlby, 1980; Bartholomew, 1990). In brief, securely attached individuals think that they are self-worthy and find others trustworthy, and they are inclined to exhibit a higher level of life satisfaction. On the contrary, individuals with insecure attachment believe that they are unworthy and negative, try to avoid social situations (emotional distance from intimate relationships), and exhibit fear of rejection. They tend to report a lower level of life satisfaction and are more likely to show reactive aggression than securely people in the same situation.

Recently, attachment styles have been evaluated based mainly on two dimensions, namely, anxiety and avoidance (Brennan et al., 1998). Thus, this involves an essential dimension of attachment, namely, attachment avoidance. Individuals who experience attachment avoidance have to gain others’ approval, maintain a significant social distance, have negative and unworthy self-model, and passively avoid close relationships (Blackwell et al., 2017; D’Arienzo et al., 2019).

As Fredrickson’s (2004) broaden-and-build theory argues, positive emotions broaden an individual’s awareness and cognition mode, flexibility, creativity, openness, and forward-looking. Over time, this expanded behavioral repertoire builds long-lasting personal resources, such as psychological resources (resilience, optimism), intellectual resources (problem-solving skills), physiological resources (physical coordination), and social resources (consolidating existing social connections, establishing new social relationships). In contrast, negative emotions, on the other hand, are narrowed an individual’s way of thinking and personal resources. In this regard, an individual with attachment avoidance will reduce the construction of the individual’s positive psychological resources and will negatively affect the individual’s healthy mental process (Raissifar and Akhavan Anvari, 2020). Against this background, attachment avoidance may play a specific intermediary role between life satisfaction and PsyCap. Life satisfaction will have a negative and significant effect on PsyCap through attachment avoidance. Thus, the following is the hypothesis.

**Hypothesis 2**: Attachment avoidance mediates between life satisfaction and PsyCap.

Individuals with attachment avoidance always have strong rejection sensitivity (Özen et al., 2011). Specifically, insecure participants have high rejection sensitivity groups. Rejection sensitivity is a disposition of the personality, mainly manifested as anxiously expecting, readily perceiving, and overreacting to rejection (Romero-Canyas et al., 2010). An individual with high rejection sensitivity, i.e., rejection sensitivity as a vulnerability factor, may balance this by reducing their investment in romantic social relationships to prevent anticipated rejection (Downey et al., 2000).

Rejection sensitivity is associated with the quality of life (Ng and Johnson, 2013). People have different perceptions and reactions to rejection. According to the cognitive-affective processing systems theory (CAPS; Mischel and Shoda, 1995), a person’s behavior varies across situations and helps explain why different situations elicit a heightened reaction. In addition, individuals with poorer quality of life and social support are also correlated with rejection sensitivity. Thus, the hypothesis is as follows.

**Hypothesis 3**: Rejection sensitivity moderates the relationships among life satisfaction, attachment avoidance, and PsyCap.

**FIGURE 1:**
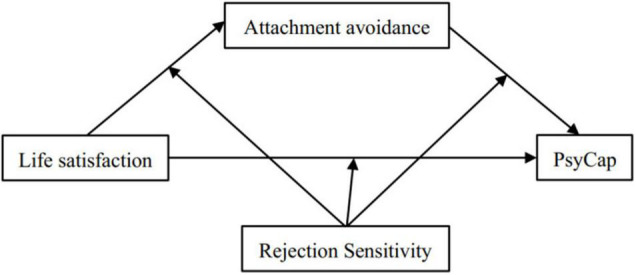
The hypothesis model of this research.

In summary, based on integrating attachment theory and CAPS theory, this study constructs a moderated mediation model from the perspective of positive psychology theory (refer to Figure 1). This study also investigates the relationship between life satisfaction, attachment avoidance, rejection sensitivity, and PsyCap. Specifically, this study aims to examine the mediating (i.e., attachment avoidance) and moderating (i.e., rejection sensitivity) mechanisms of life satisfaction in predicting the level of PsyCap. Meanwhile, it guides promoting college students to enhance PsyCap, weaken rejection sensitivity, correctly understand attachment avoidance, and improve life satisfaction.

## Materials and Methods

### Participants

All data were collected *via* an online questionnaire. A simple random sample of 1,232 students was used for the assessment, and the respondents were college students in China. After eliminating invalid questionnaires (questionnaires that indicate the answering time was less than 240 s and more than 1,800 s), a total of 999 valid questionnaire data were collected (334 Male; 665 Female; M ± SD = 19.45 ± 1.21).

### Measures

#### The Satisfaction With Life Scale

The Satisfaction with Life Scale has five items rated on a 7-point Likert-type scale ranging from 1, disagree entirely, to 7, completely agree. Cognitive-judgmental aspects were used based on the subjects (Diener et al., 1985). The scale of this study has a high Cronbach’s α value of 0.864.

#### Experiences in Close Relationship Scale

The Chinese version of the Experiences in Close Relationship (ECR) Scale measures a total of 36 items (18 items for anxiety dimension and 18 items for avoidant dimension) to assess the attachment style (Li and Kato, 2006). Each item was rated on a 7-point Likert-type scale ranging from 1, disagree, to 7, agree entirely. Odd-numbered items were scored on the attachment avoidance dimension. The Cronbach’s α value for this study was 0.939, and the Cronbach’s α value for the attachment avoidance dimension was 0.893.

#### Positive Psychological Capital Scale

The Positive Psychological Capital Scale consists of four dimensions, namely, self-efficacy, resilience, hope, and optimism, with 26 items (Zhang et al., 2010). Each topic was evaluated on a 7-point Likert-type scale ranging from 1, disagree, to 7, agree entirely. The higher the subjects’ scores, the better their positive PsyCap status. The Cronbach’s α value for this study was high at 0.950.

#### Rejection Sensitivity Scale

The Rejection Sensitivity Scale consists mainly of 18 situations in which college students need help from others in their daily life (Downey and Feldman, 1996; Zhao et al., 2012). The subjects’ response to each situation is composed of two dimensions: the degree of anxiety about rejection and the expected degree of acceptance. Each item in the degree of anxiety about rejection dimension was rated on a 6-point Likert-type scale ranging from 1, not worried at all, to 6, very worried. The higher the subjects’ scores, the higher the anxiety and worry about rejection. Each item of the expected degree of acceptance dimension was rated on a 6-point Likert-type scale ranging from 1, completely impossible, to 6, very likely. The higher the subjects’ scores, the higher the possibility that others will accept their request. The Cronbach’s α value of this study was 0.938.

### Procedure

All data collected were recorded on a computer and processed using SPSS Statistics version 23.0 and AMOS version 22.0. Harman’s factor analysis was used for common method bias, and Pearson’s correlation was used to explore the correlation between the main variables. Meanwhile, we used the multiple regression method to examine the mediation and moderation effects and path analysis. To avoid possible skewness problems, the calculation of the structural equation model (SEM) adopts the bootstrapping method (Preacher et al., 2007).

### Statistical Analyses

First, descriptive and correlation statistics were conducted using IBM SPSS version 23.0. Second, the mediation effect was examined using Hayes’s PROCESS macro (Model 4). Third, the full model was examined using Hayes’s PROCESS macro (Model 59). The moderated mediation model was verified using the AMOS version 22.0 full model test. We used the bootstrapping method to determine whether the significance of the mediation effects is according to a moderator’s value.

## Results

### Common Method Bias

Two common method biases, including process control and Harman’s single-factor test, are used. Using the questionnaire, we adhere to the principles of anonymity and confidentiality and the data results are only used for academic research. Harman’s single-factor test found that 16 factors had eigenvalues greater than 1, and the first factor of the amount of variation explained was 16.313%, far less than 40% of the critical criterion. Thus, the common method bias in this study was not so strong to influence the relationship between variables.

### Descriptive and Correlation Statistics

Table 1 indicates the leading research variables’ means, SDs, and correlations. Correlation analyses demonstrated that life satisfaction was positively associated with PsyCap and significantly negatively associated with attachment avoidance and rejection sensitivity. PsyCap was significantly negatively associated with both attachment avoidance and rejection sensitivity. Attachment avoidance has an incredibly positive relationship with rejection sensitivity.

**TABLE 1:** Means, standard deviations, and correlations.

Variables	M ± SD	1	2	3	4
1 Attachment avoidance	3.591 ± 0.668	1			
2 Rejection sensitivity	4.291 ± 1.747	0.308**	1		
3 PsyCap	4.690 ± 0.731	−0.265**	−0.417**	1	
4 Life satisfaction	4.258 ± 1.091	−0.188**	−0.339**	0.437**	1

**p < 0.01.

### Results of the Mediating Effect of Attachment Avoidance

This study uses the SPSS PROCESS macro (Model 4) by Hayes (2012) to assess attachment avoidances’ mediating effect in the links to life satisfaction on PsyCap. All data are processed and converted into *Z*-scores. The results (refer to Tables 2, 3) indicate that life satisfaction has a significant predictor of PsyCap (β = 0.293, *t* = 15.330, *p* < 0.01), with 95% CI of [0.255, 0.330]. In addition, the direct predictive effect of life satisfaction on PsyCap remains significant when mediating variables are included (β = 0.269, *t* = 14.130, *p* < 0.01), with 95% CI of [0.231, 0.306].

**TABLE 2:** The mediation model of attachment avoidance.

Regression equation (*N* = 999)		Fitting index			Coefficient significance	
		
Outcome variable	Predictor variable	*R*	*R* ^2^	*F (df)*	β	*t*
PsyCap		0.437	0.191	235.012 (1) **		
	Life satisfaction				0.293	15.330**
Attachment avoidance		0.188	0.035	36.397 (1) **		
	Life satisfaction				−0.115	−6.033**
PsyCap		0.475	0.226	144.969 (2)**		
	Attachment avoidance				−0.207	−6.681**
	Life satisfaction				0.269	14.130**

All variables in the model are standardized and brought into the regression equation, as follows. **p < 0.01.

**TABLE 3:** Analysis of Total effect, direct effect, and mediating effect.

	Effect	Boot SE	Boot LLCI	Boot ULCI	Percentage of effect value
Total effect	0.293	0.020	0.000	0.255	
Direct effect	0.269	0.019	0.000	0.231	91.8%
Mediating effect of attachment avoidance	0.024	0.006	0.013	0.037	8.2%

Life satisfaction has a significant negative predictive effect on attachment avoidance (β = −0.115, *t* = −6.033, *p* < 0.01), with 95% CI of [−0.152, −0.078]. Attachment avoidance also has a significant negative predictive effect on PsyCap (β = −0.207, *t* = −6.681, *p* < 0.01), with 95% CI of [−0.268, −0.146]. Furthermore, the upper and lower 95% bootstrapping CI for the direct effect of life satisfaction on PsyCap and the mediating effect of attachment avoidance did not contain 0 (refer to Table 3). This study suggests that life satisfaction directly predicts PsyCap through the mediating effect of attachment avoidance. The direct effect was 0.269, and the mediating effect was 0.024. They accounted for 91.8 and 8.2% of the total effect (0.293), respectively.

### Results of the Moderation Mediating Model

Hayes’s PROCESS macro (Model 59) assumes that the moderator affects all three paths of the mediated model, consistent with the theoretical model. The moderated mediation model of this study was examined using Model 59. The results (refer to Table 4) showed that rejection sensitivity has significantly moderating effect between life satisfaction and attachment avoidance (β = 0.049, *t* = 2.793, *p* < 0.01), with 95% CI of [0.014, 0.083]. Rejection sensitivity has no significant moderating effect between attachment avoidance and PsyCap (β = 0.012, *t* = 0.422, *p* > 0.01), with 95% CI of [−0.044, 0.068] and between life satisfaction and PsyCap (β = −0.006, *t* = −0.348, *p* > 0.01), with 95% CI of [−0.041, 0.029]. This result suggests that rejection sensitivity can only play a moderating role in predicting attachment avoidance by life satisfaction.

**TABLE 4:** The moderated mediation model analysis.

Regression equation (*N* = 999)		Fitting index			Coefficient significance	
		
Outcome variable	Predictor variable	*R*	*R* ^2^	*F (df)*	*B*	*T*
Attachment avoidance		0.331	0.110	40.914 (3)**		
	Life satisfaction				−0.061	−3.134**
	Rejection sensitivity				0.179	0.214**
	Life satisfaction × Rejection sensitivity				0.049	2.793**
PsyCap		0.535	0.286	79.602 (5)**		
	Attachment avoidance				−0.127	−3.950**
	Life satisfaction				0.216	11.189**
	Rejection sensitivity				−0.198	0.022**
	Attachment avoidance × Rejection sensitivity				0.012	0.422
	Life satisfaction × Rejection sensitivity				−0.006	−0.348

**p < 0.01.

**FIGURE 2:**
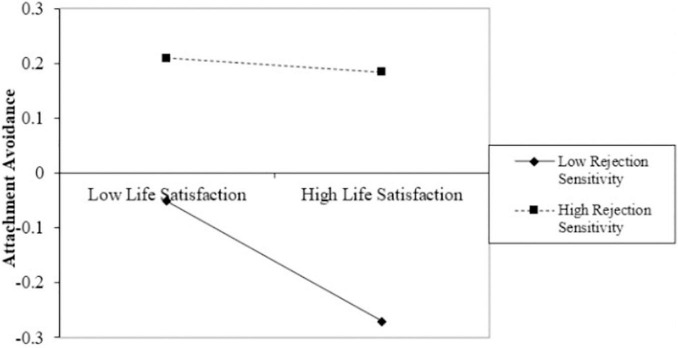
The rejection sensitivity moderates life satisfaction and attachment avoidance.

To reveal more clearly how rejection sensitivity moderates the relationship between life satisfaction and attachment avoidant, rejection sensitivity was divided into high and low groups by M ± 1 SD using SPSS, and simple slope tests were performed. A further simple slope plot (Hayes and Matthes, 2009) indicated that the life satisfaction of individuals with low levels of rejection sensitivity is a stronger predictor of attachment avoidance than individuals with high rejection sensitivity (refer to Figure 2). Specifically, for individuals with low rejection sensitivity (M − 1 SD), life satisfaction had a significant negative predictive effect on attachment avoidance (simple slope = −0.061, *t* = −3.140, *p* < 0.001). While for individuals with high rejection sensitivity (M + 1 SD), the negative predictive effect of life satisfaction on attachment avoidance tends to be diminished (simple slope = 0.049, *t* = 2.793, *p* < 0.001) (refer to Table 5).

**TABLE 5:** Direct effects on different levels of rejection sensitivity.

Rejection sensitivity		Effect	Boot SE	Boot LLCI	Boot ULCI
Direct effect	−1 (M−1 SD)	–0.110	0.027	0.168	0.276
	0 (M)	–0.061	0.019	0.178	0.253
	1 (M+1 SD)	–0.012	0.025	0.161	0.259
The mediating role of attachment avoidance	−1 (M−1 SD)	0.015	0.007	0.004	0.031
	0 (M)	0.008	0.004	0.002	0.016
	1 (M+1 SD)	0.001	0.003	–0.005	0.009

The omnibus test of the conditional indirect effect (IE) suggested that rejection sensitivity significantly moderated the IE of life satisfaction on attachment avoidant as the 95% CI of [0.137, 0.221]. The IE was significant at M − 1 SD with IE = −0.110, with 95% CI of [−0.163, −0.057], and the mean with IE = −0.061, with 95% CI of [−0.096, −0.023]. The IE was not significant at the M + 1 SD with IE = −0.012, with a 95% CI of [−0.062, 0.037].

The above results found that attachment avoidance mediates between life satisfaction and PsyCap. Rejection sensitivity moderates the relationship between life satisfaction and attachment avoidance. This study indicates that PsyCap, attachment avoidance, rejection sensitivity, and life satisfaction may constitute a moderated mediation model. To verify the hypothetical model, AMOS version 22.0 was used to conduct the full model test. The bootstrapping method was used to calculate the SEM on a selected sample size of 5,000, which showed that the effect was significant when the 95% CI did not include 0.

**FIGURE 3:**
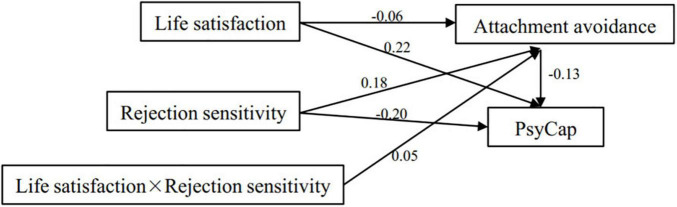
The results of moderating mediating model.

The results (refer to Figure 3) reveal that the direct effect of life satisfaction on attachment avoidance was significant, with 95% CI of [−0.10, −0.02], *p* < 0.01. The IE of rejection sensitivity on the results (refer to Figure 3) reveal that the direct effect of life satisfaction on attachment avoidance was significant, with 95% CI of [−0.10, −0.02], *p* < 0.01. The IE of rejection sensitivity on PsyCap was significant, with 95% CI of [−0.25, −0.15], *p* < 0.01. The direct effect of rejection sensitivity on attachment avoidance was significant, with 95% CI of [0.13, 0.22], *p* < 0.01. Rejection sensitivity × life satisfaction had a significant direct effect on attachment avoidance with 95% CI of [0.01, 0.09], *p* < 0.01. The IE of life satisfaction on PsyCap was significant, with 95% I of [0.17, 0.26], *p* < 0.01. The direct effect of attachment avoidance on PsyCap was significant, with 95% CI of [0.22, 0.35], *p* < 0.01.

The model fitting indices of the SEM were χ^2^/df = 3.19, goodness-of-fit index (GFI) = 0.997, comparative fit index (CFI) = 0.992, incremental fit index (IFI) = 0.992, adjusted goodness-of-fit index (AGFI) = 0.981, normed fit index (NFI) = 0.989, Tacker-lewis index (TLI) = 0.962, root mean square error of approximation (RMSEA) = 0.047, and root mean square residual (RMR) = 0.027. According to the good fitting criteria (Hou et al., 2004), the values were χ^2^/df < 5, RMSEA < 0.08, and NFI, CFI, and GFI > 0.90, indicating that the model is well-fitted.

## Discussion

Based on previous research, attachment theory, and CAPS theory, this study constructs a moderated mediation model from the perspective of positive psychology theory. This model not only clarifies the question of “How life satisfaction affects” PsyCap but also responds to the question of under what conditions life satisfaction had a more significant impact on PsyCap. This study has specific theoretical significance for deepening the relationship between life satisfaction and individual PsyCap.

### Mediating Effects of Attachment Avoidance

Attachment influences an individual’s PsyCap in a complex way, and attachment avoidance plays a critical role as an explanatory dimension of attachment style. This study explores the impact of life satisfaction, attachment avoidance, and rejection sensitivity on PsyCap. The investigation of mediating effect in this study reveals that life satisfaction could predict PsyCap through the mediating role of attachment avoidance, and the mediating effect accounted for 8.2% of the total effect. This result presents that part of the major impact of life satisfaction on PsyCap is a direct effect, and the other part is through the mediator of attachment avoidance, which is consistent with Hypotheses 1 and 2.

Notably, the direct effect of life satisfaction on PsyCap is positive, whereas the effect of attachment avoidance through the intermediary item on PsyCap is negative. This point shows that life satisfaction and PsyCap are not a simple linear relationship. In other words, the higher life satisfaction of an individual does not mean that their PsyCap is strong. Lower life satisfaction and more vital attachment avoidance traits lead to lower PsyCap construction. Therefore, the result of the mediator shows that the relation to attachment avoidance and PsyCap has two sides.

First, PsyCap also increases and constructs individuals with higher life satisfaction more quickly. PsyCap is an emerging core higher-order construct encompassing psychological resources such as optimism, self-efficacy, resilience, and hope. These psychological capacities are more substantially linked with life satisfaction (Bailey et al., 2007; Tagay et al., 2016; Martínez-Martí and Ruch, 2017).

Second, the negative mediating effect of attachment avoidance reduces the construction of PsyCap. The positive impact between life satisfaction and PsyCap is emphasized, but we cannot ignore the adverse effect between the two. Attachment avoidance is a crucial characteristic of an insecure attachment style. Drawing on attachment theory, contrary to those who have insecure attachments, those individuals with secure attachment tend to have more positive self-evaluation and others’ evaluations and feel good about themselves, and trust others. Individuals with insecure attachments have lower levels of hope (Shorey et al., 2003).

Furthermore, this study also reveals a positive correlation between life satisfaction and PsyCap, which validates previous related studies (Liao et al., 2017; Choi et al., 2018; Bajwa et al., 2019). Individuals with higher life satisfaction adjust their attachment avoidance, weaken the rejection sensitivity, and enhance the construction of PsyCap in a spiral process. This view is also more consistent with the broaden-and-build theory.

### Moderating Effect of Rejection Sensitivity

This study examines the relationship of rejection sensitivity among life satisfaction, attachment avoidance, and PsyCap to verify Hypothesis 3. This study shows that rejection sensitivity moderates the relationship between life satisfaction and attachment avoidance. Rejection sensitivity does not moderate life satisfaction and PsyCap. In addition, rejection sensitivity also does not moderate attachment avoidance and PsyCap. Thus, Hypothesis 3 is partially proven.

To be more specific, this study shows that rejection sensitivity moderates the relationship between life satisfaction and attachment avoidance. Individuals with high rejection sensitivity using avoidant strategy tend to have high attachment avoidance (Feldman and Downey, 1994; Scharf et al., 2014). This viewpoint showed that it is a risk factor for undermining life satisfaction and reducing PsyCap. It seems that providing interventions to improve attachment avoidance may be helpful for high rejection sensitivity individuals. Furthermore, rejection sensitivity was added to the defensive motivation system (DMS) (Downey et al., 2004). The DMS “helps” individuals with a high level of rejection sensitivity quickly detect rejection and react toward rejection or ambiguous clues in social situations. When faced with negative stimuli or frustration, the DMS is activated to protect oneself from possible danger. Therefore, this regulatory effect may be that individuals with high rejection sensitivity tend to have personality traits of high attachment avoidance not influenced by life satisfaction.

This study reveals that rejection sensitivity does not moderate life satisfaction and PsyCap. According to the CAPS theory, the differences exhibited by individuals in different situations reflect a stable and organic internal personality structure. The viewpoint suggests that there may be both temporary and persistent cases of rejection sensitivity.

In addition, this study has many limitations. Given the cross-sectional design of this study, issues concerning causal direction are left open and need to be explored further in a longitudinal or experimental study. The other limitation is that the empirical research paradigms that manipulate independent or mediating variables can be used to examine the effects of life satisfaction on individual PsyCap to explore the mechanisms involved deeply. Despite the limitations, this study reveals a moderated mediation model of the relationship between life satisfaction and PsyCap. It provides a deeper cognitive explanation of the internal mechanism between life satisfaction and PsyCap.

## Conclusion

This study makes the following conclusions:

(1) Life satisfaction has a significant positive predictive effect on PsyCap.
(2) Attachment avoidance mediates life satisfaction and PsyCap.
(3) Rejection sensitivity moderates the relationship between life satisfaction and attachment avoidance. Specifically, compared to individuals with high rejection sensitivity, this mediated pathway works more for low rejection sensitivity individuals.

## Data Availability Statement

The original contributions presented in the study are included in the article/supplementary material, further inquiries can be directed to the corresponding author/s.

## Ethics Statement

Ethical review and approval was not required for the study on human participants in accordance with the local legislation and institutional requirements. Written informed consent was obtained from all participants for their participation in this study.

## Author Contributions

HS and ZI conceived the research. HS analyzed the data, tables, and figures, and drafted the initial manuscript. ZI and LF provided critical edits. All authors discussed the results and contributed to the final manuscript.

## Conflict of Interest

The authors declare that the research was conducted in the absence of any commercial or financial relationships that could be construed as a potential conflict of interest.

## Publisher’s Note

All claims expressed in this article are solely those of the authors and do not necessarily represent those of their affiliated organizations, or those of the publisher, the editors and the reviewers. Any product that may be evaluated in this article, or claim that may be made by its manufacturer, is not guaranteed or endorsed by the publisher.
